# Let’s Connect to Keep the Distance: How SMEs Leverage Information and Communication Technologies to Address the COVID-19 Crisis

**DOI:** 10.1007/s10796-021-10210-z

**Published:** 2021-10-13

**Authors:** Charlotte Wendt, Martin Adam, Alexander Benlian, Sascha Kraus

**Affiliations:** 1 Institute of Information Systems and Electronic Services, Technical University of Darmstadt, Hochschulstr. 1, 64289 Darmstadt, Germany; 2 Faculty of Economics and Management, Free University of Bolzano, Piazza Università 1, 39100 Bolzano, Italy

**Keywords:** Technology-organization-environment framework, Technology-affordances-and-constraints theory, Information and communication technologies, SME, Crisis response, COVID-19

## Abstract

COVID-19 caused significant challenges for small and medium-sized enterprises (SMEs) in the event industry. To address these challenges, many SMEs leveraged information and communication technologies (ICTs), with some even emerging strengthened from the crisis. Drawing on the technology-organization-environment framework and technology-affordances-and-constraints theory, we investigate the adoption of ICTs as a crisis response strategy in 10 SMEs in the German business event (e.g., corporate events, conferences) industry. Our findings reveal that ICT adoption not only depends on rational decisions based on organizational, environmental, and technological characteristics, but also on these dimensions’ interrelationship and the specific ICTs’ affordances and constraints. Introducing readily available ICTs (e.g., video-conferencing) has significant potential in addressing physical distancing in the short and medium term, while more sophisticated ICTs (e.g., virtual reality) are more likely to gain importance in the long term. Thus, we expand our understanding of organizational technology adoption and ICT-enabled crisis response strategies in SMEs.

## Introduction

Small and medium-sized enterprises (SMEs) in the event industry, such as live-communication agencies and conference organizers, bring people together and provide a space for communication and knowledge sharing, thus fulfilling the human need for interaction, connection, and relatedness (Getz & Page, 2016; Seraphin, 2020). Events offered by these SMEs attribute their popularity to the unique emotional and experience-oriented environment they create, which is often prevalent in business events (e.g., corporate events, conferences), the event industry’s main revenue source (Feuerbach et al., 2020; Richards & Jarman, 2021). Accordingly, SMEs in the business event industry naturally provide a platform for networking, conversation, and the development of trust-based relationships among its customers (i.e., enterprises paying for attending their offerings), which is best realized in personal and physical locations or encounters (Richards & Jarman, 2021).

Given their high dependency on physical interactions amongst business people (Seraphin, 2020), it is not surprising that many SMEs in the business event industry were — and still are — severely affected by the economic consequences of the COVID-19 pandemic (Allied Market Research, 2021; ICCA, 2020). Measures taken by many governments to mitigate the spread of the virus (e.g., physical distancing,^1^ travel restrictions, and wearing masks in public) limit physical interaction among people to the absolute minimum (Clark et al., 2020; Ferguson et al., 2020; Kraus et al., 2020a) and forced SMEs in the business event industry to cancel or postpone a multitude of events. Moreover, many of its customers faced financial uncertainty themselves, which resulted in reduced participant numbers, additional event cancellations, and losses in revenues (GCB, 2020). Consequently, the global event industry, which was valued at more than 1 trillion USD in 2019, experienced a 30% decline in revenue in 2020 and there is a similar forecast for 2021 (Allied Market Research, 2021; UFI, 2021) creating an existential threat for many SMEs in the business event industry (Dimson et al., 2020). Despite these challenges, some SMEs have not only survived, but have become stronger, for instance by quickly switching from organizing in-person events to offering holistic support for virtual events and thereby, even increasing the size and diversity of their audiences (AirLST, 2021). Understanding their success factors is important to deriving insights for other SMEs in the business event industry to produce suitable responses to the current and future crises.

Taking into account the COVID-19-related regulations, SMEs in the business event industry had two options: either adhering to physical distancing and related guidelines (e.g., wearing face masks) during physical events, or adopting information and communication technologies (ICTs) and thereby innovating their business models by transitioning to hosting virtual events. While the former has been successfully applied by some enterprises in other industries (e.g., supermarkets, retail, pharmacies), SMEs in the business event industry risk to forfeit their attractiveness for their customers because adhering to these guidelines removes significant event value due to the drastic reduction in emotional connection which is an integral part of the event (Dennis & Valacich, 1999). For instance, physically attending a music festival or celebrating an enterprise’s anniversary while keeping a certain distance to one’s friends does not create an atmosphere conducive to building relationships and connections (Eagle et al., 2009). Additionally, not being able to view the sales person’s facial expression behind the face mask while negotiating at a fair does not create the foundation of trust required to sign a contract that involves a substantial investment (Olivola et al., 2014; Oosterhof & Todorov, 2008). However, the latter option also has challenges associated with it. Even though ICTs would be ideal for overcoming physical distancing constraints, the business event industry has largely been immune to digitization initiatives prior to the outbreak of the COVID-19 pandemic, with most initiatives limited to items such as the use of mobile applications as part of business events (Schreiber et al., 2019) or virtual reality environments offered for business-related team events (VArea, 2020).

Prior research on crisis response strategies has primarily focused on how crises encourage enterprises of different sizes and industries to adopt ICTs as well as to innovate their business models to overcome a crisis (e.g., Gkeredakis et al., 2021; Majchrzak et al., 2016; Wenzel et al., 2020). However, finding and implementing an appropriate strategic response to the COVID-19-induced crisis by adopting ICTs may require a tailored approach for SMEs in the business event industry, and thus warrants research attention as specified below.

First, SMEs are known for experiencing significant challenges when adopting ICTs as they often struggle to attract and retain the required skills and expertise, and are usually less aware of available ICTs and their benefits (e.g., Chan et al., 2020; Thong, 1999). Moreover, they often cannot afford expensive, customized ICT solutions as they generally have limited financial resources (Chan et al., 2020; Heidt et al., 2019). Additionally, these financial resources have typically been depleted with the outbreak of the COVID-19 pandemic and the related decline in revenues that many SMEs in the business event industry have experienced (Dimson et al., 2020; UFI, 2021). However, using ICT adoption as a lever to innovate an SME’s business model requires financial as well as human resources (e.g., technological expertise) to handle substantial changes of the enterprise’s value proposition and value creation (Breier et al., 2021; Foss & Saebi, 2017; Kraus et al., 2020b). Even though SMEs’ specific challenges related to ICT adoption are well-known, they are under-investigated by the majority of information systems (IS) scholars (e.g., Chan et al., 2020; Li et al., 2017). Therefore, it is currently unclear whether and how ICT adoption can promote what is normally a resource-intense innovation of an enterprise’s business model, and whether such a strategic response to the crisis is appropriate for SMEs in the business event industry. As such, we propose the following first research question:

- RQ1: How can ICT adoption serve as a strategic crisis response for SMEs in the business event industry to address the COVID-19-induced crisis?

Second, one significant but under-explored aspect of ICT-enabled business model innovation (BMI) is the use of specific ICTs to overcome physical distances and to enable entrepreneurial activities in areas that have traditionally been dominated by physical interaction, such as the business event industry (Seraphin, 2020; Yeoman, 2013). Previous IS research has extensively studied the role of various ICTs in digital transformation and has also investigated how various ICTs have acted as facilitators to cope with societal and organizational challenges by examining the affordances and constraints of various ICTs (e.g., Majchrzak et al., 2016; Volkoff & Strong, 2013). For instance, Essén and Värlander (2019) investigated the impact of patient self-registration services in hospitals on institutional change and Selander and Jarvenpaa (2016) researched the impact of social media on supporters of social movement organizations. In addition, technology and innovation adoption by organizations has been researched through the lens of the technology-organization-environment (TOE) framework (e.g., DePietro et al., 1990; Seethamraju, 2015; Zhu et al., 2004). Yet, the COVID-19-specific environmental disruptions, arising from measures imposed by governments (i.e., mandatory physical distancing and travel bans) as well as from changing market requirements (i.e., lacking possibility to adhere to such guidelines without losing attractiveness for customers) are putting SMEs in the business event industry under enormous pressure to identify and implement appropriate ICTs. Thus, there is a lack of understanding what ICTs can be adopted by SMEs in the business event industry to allow for BMI under high time pressure and to be able to connect event participants while keeping the required distance. Therefore, we ask the following second research question:

- RQ2: What ICTs are most promising for SMEs in the business event industry to adapt to the COVID-19-related physical distancing guidelines?

To answer these research questions, we build upon previous research on strategic crisis response strategies (Kraus et al., 2020a; Wenzel et al., 2020) as well as on the TOE framework (DePietro et al., 1990) to investigate SME-specific internal and external factors that are required to successfully and sustainably adopt ICTs in response to the COVID-19-induced crisis. Furthermore, we draw on technology-affordances-and-constraints theory (Majchrzak et al., 2016) as a theoretical lens through which we elaborate the benefits and obstacles of ICTs, thereby investigating their particular relevance for SMEs in the business event industry to connect people, whilst also adhering to physical distancing guidelines.

Our findings contribute to existing research on organizational ICT adoption and ICT-enabled crisis response strategies as it relates to SMEs in three important ways: First, by uncovering and contrasting the affordances and constraints of the most promising ICTs in the context of physical distancing, we contribute to an enhanced understanding of the impact of such ICTs’ affordances and constraints on ICT adoption by SMEs. Second, by shedding light on the impact of disruptive environmental changes (i.e., the outbreak of a pandemic) on the TOE framework’s dimensions, we provide a better understanding of the dimensions’ characteristics and interrelationships, and thereby contribute to a more holistic view of the TOE framework in the context of SMEs. Third, we contribute to literature on ICT-enabled BMI as strategic crisis response by uncovering how SMEs respond to the crisis, taking into account their specific characteristics. Moreover, we reveal practical implications for SMEs in the business event industry by providing guidance on how to select appropriate ICTs for innovative event structures based on affordances and constraints, which is required to address the challenges associated with the current crisis and to be well prepared to overcome future crises.

## Theoretical Background

### ICT-Enabled Business Model Innovation as a Crisis Response Strategy

According to previous research on crisis response strategies, innovating an enterprise’s business model is a promising approach to overcome a crisis in the long run and comprises measures such as alternative usage of resources, digitalization of workflows, and business model adjustments (Kraus et al., 2020a; Wenzel et al., 2020). Such a "process of designing a new, or modifying the firm’s extant activity system", is defined as BMI (Amit & Zott, 2010, p. 2) and is crucial to generating long-term competitive advantage, adjusting to market changes or to even surviving in the market. Therefore, BMI is characterized by substantial changes in the enterprise’s value proposition and value creation (Breier et al., 2021; Kraus et al., 2020b), and requires active initiation of change which again involves financial and human resources as well as changes in existing behavior patterns (Foss & Saebi, 2017; Goduscheit et al., 2021).

Moreover, previous IS research explored the role of different ICTs in digital transformations and as a facilitator to cope with societal challenges of various kinds (see Majchrzak et al., 2016 for an overview). In the context of the COVID-19 pandemic, previous research investigated how the induced crisis encouraged enterprises to adopt ICTs and how ICTs can be leveraged to enhance productivity and competitiveness to secure business continuity (Gkeredakis et al., 2021; Kraus et al., 2020a; Papadopoulos et al., 2020). Accordingly, ICT adoption enables enterprises to prepare for organizational and environmental challenges by shifting organizational practices to digital spaces, and by allowing for work continuity and acceleration of innovation (Gkeredakis et al., 2021; Papadopoulos et al., 2020).

Yet, even though the role of ICT adoption in securing business continuity during crises is acknowledged, there is limited guidance for SMEs on how to prepare for ICT adoption, what barriers and drivers for adoption to consider and how to choose and sustainably implement ICTs (Gkeredakis et al., 2021; Papadopoulos et al., 2020).

### Technology-Organization-Environment Framework

#### Technology-Organization-Environment Framework as an Overarching Framework for ICT Adoption

To elaborate how ICT adoption can act as a crisis response strategy, a more nuanced understanding of ICTs and their adoption by SMEs is required, using the TOE framework. The TOE framework encompasses three distinct dimensions that influence an organization’s intent to adopt a technology, as well as the adoption process itself (DePietro et al., 1990): First, the *technological dimension* includes characteristics and availability of technologies that are either already used by an enterprise or generally available in the market. Second, the *organizational dimension* refers to descriptive characteristics such as the enterprise’s size, (slack) resources, managerial structure, and communication processes which can either foster or impede a technology’s adoption. Third, the *environmental dimension* comprises factors in the enterprise’s periphery such as governmental regulations, characteristics of the enterprise’s industry, the market structure as well as the access to technology support infrastructure.

**Fig. 1:**
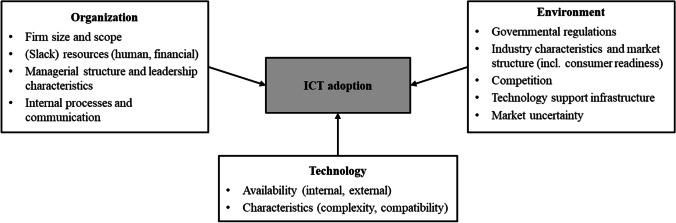
TOE framework applied to ICT adoption (DePietro et al., 1990; Thong, 1999; Zhu et al., 2004)

The TOE framework has been studied in a variety of IS domains (Baker, 2012; Zhu et al., 2004). For instance, it has been deployed to investigate the adoption of electronic businesses (Zhu et al., 2004) as well as to advance our understanding of the adoption of open systems (Chau & Tam, 1997) and IS applications (Thong, 1999). Figure 1 illustrates the TOE framework’s dimensions and frequently analyzed sub-dimensions.

Given its properties, the TOE framework is highly suitable to be applied in our research as an overarching framework to investigate ICT adoption by SMEs in the business event industry in response to the COVID-19-induced crisis for two main reasons: First, our research investigates technology adoption on an organizational, rather than on an individual level. The TOE framework is one of the most popular and frequently employed concepts when investigating technology and innovation adoption on such an organizational level (Baker, 2012; Zhu et al., 2004). Second, our research comprises the COVID-19-induced changes in the environment of SMEs in the business event industry which also impacts the organizational and technological dimension. While other theories and frameworks with a focus on organizational technology adoption (e.g., the diffusion of innovation theory by Rogers, 2003) concentrate on the intraorganizational context, the environmental dimension is an essential part of the TOE framework which makes it particularly suitable for our research.

However, the framework’s dimensions require adaptation to the specific environmental and organizational context to investigate ICT adoption by SMEs in the business event industry in the specific context of the COVID-19 pandemic. Specifically, the order of the three dimensions is adjusted to acknowledge that the pandemic affected the environmental dimension the most, followed by the organizational dimension, while the technological dimension is the least affected: The *environmental dimension* (i.e., business event industry and its market) has been disrupted by changing circumstances. Still, it is unclear how this disruption affects ICT adoption, which will be explored in more detail as part of our analysis. By contrast, the *organizational dimension* (i.e., SMEs) describes the specific characteristics that are largely determined by the organizational size itself and will be outlined below, while the consequences of such characteristics on ICT adoption will form part of our analysis. With respect to the *technological dimension* (i.e., ICTs), technology availability was significantly impacted by the pandemic. To consider and to compare the impact of different types of ICTs, we introduce a technology’s affordances and constraints as a complementary concept to better understand the underlying causes of successful ICT adoption.

#### Organizational Specificities of Small and Medium-Sized Enterprises

Previous IS research emphasized the fundamental structural differences between SMEs and large enterprises and the impact of specific SME characteristics on technology adoption and related implementation barriers (e.g., Arendt, 2008; Ballantine et al., 1998; Chang, 2020). In this context, the *digital divide* refers to the fact that SMEs often lag opportunities to access and appropriately use ICTs due to the differentials in company size (Arendt, 2008; Wielicki & Arendt, 2010). The reason for this disparity lies in the SMEs’ internal characteristics which can be further divided into organizational and leadership characteristics, which are directly linked to SMEs’ small number of employees (Heidt et al., 2019). Organizational challenges typically comprise limited financial resources (i.e., a small asset base and general cash flow difficulties), lower formalization levels, multiple-role identities, and geographical insularity (e.g., Heidt et al., 2019; Riemenschneider et al., 2003). In addition, difficulties in accessing appropriate skills have been uncovered to be one of the main reasons why SMEs struggle to adopt and to take advantage of ICTs and as such, exacerbate the digital divide between SMEs and larger enterprises (Arendt, 2008).

However, organizational characteristics of SMEs typically also include a culture with flat hierarchies, short communication channels, fast decision-making, and high levels of trust (Cragg et al., 2011; Huang et al., 2010). Moreover, SMEs usually have less legacy information technology (IT) infrastructure than larger enterprises and can avoid investment costs of on-premise solutions by accessing packaged solutions from third-party service providers (Huang et al., 2010; Seethamraju, 2015). Thus, SMEs can adopt affordable or even free ICTs to minimize costs. For instance, Chan et al. (2020) examine how SMEs can use commonly available technologies such as *Skype* and *Google Cloud* to develop business opportunities. The results demonstrate that such ICTs enable leading-edge SMEs to uncover industry patterns, engage with stakeholders, involve external resources, evaluate product performance and market needs, as well as upgrade internal processes.

Many of the leadership characteristics of SMEs are typically shaped by the "influential role of owner-managers since they are often the prime and sole decision-maker in every operational and strategic business aspect all while being almost exclusively responsible for the survival of the enterprise" (Heidt et al., 2019, p. 1291). Such leadership characteristics comprise managerial skills, founder reputation, knowledge on IS and IT, attitude and values, as well as a strategic outlook (Heidt et al., 2019; MacGregor & Vrazalic, 2005; Mahto & Khanin, 2013). Therefore, the owner-manager’s characteristics can strongly determine an SME’s technology and innovation adoption behavior, its long-term strategy, and its investment decisions.

### Technology-Affordances-and-Constraints Theory

The concept of affordances originates in ecological psychology (Gibson, 1977) and has been successfully applied in IS research (e.g., Majchrzak et al., 2016; Markus & Silver, 2008; Volkoff & Strong, 2013). According to ecological psychology, individuals perceive the affordances of an object, rather than the object’s properties (Gibson, 1977; Michaels & Carello, 1981). For instance, a person does not perceive a bench as a wooden construction, but as a place to sit or lay down, or in other words, the bench affords sitting to one person, while it affords lying down to another person. Similarly, technologies can be regarded as sets of affordances and constraints that are not necessarily identical for different actors which explains why a specific technology is used differently by different actors and in different contexts (Leonardi, 2011; Majchrzak et al., 2016). Accordingly, affordances and constraints describe the potential interaction between actors (i.e., organizations or individual technology users) and a technology (e.g., a particular ICT), and hence are relational concepts rather than properties of actors or technologies (Majchrzak & Markus, 2013).

Previous research applied the concept of affordances to different IS contexts to theorize ICT-associated change in organizations (Majchrzak et al., 2016; Volkoff & Strong, 2013). For instance, it has been deployed to investigate the affordances associated with the implementation of an enterprise system (Volkoff & Strong, 2013), a videoconferencing technology (Li et al., 2020), patient self-registration services (Essén & Värlander, 2019), or scenario technique as an organizational strategy and foresight tool (Tiberius, 2019). In doing so, research has shown that multiple affordances can be supported by one technology and that depending on an individual’s goals, different individuals enact different affordances from one and the same technology (Leonardi, 2011). Moreover, causes for technology implementation difficulties could be identified based on affordances: So-called constraints potentially deter technology implementation and arise from the absence or inappropriate actualization of a desired affordance or from individuals that are unwilling or unable to actualize an affordance (Volkoff & Strong, 2013).

However, there is limited understanding of the affordances and constraints of different ICTs that can be applied by SMEs in the business event industry to overcome physical distancing.

## Research Methodology

### Research Design

To answer our research questions, we followed a *key informant approach* and conducted qualitative expert interviews with executives of ten SMEs in the business event industry (Huber & Power, 1985; Lechner et al., 2006). The inclusion of multiple perspectives from different interview cases ensures robustness, allows for more generalizable findings and adds confidence to findings while facilitating the exploration of the "how" and "why" that underlies the observed phenomena (Eisenhardt & Graebner, 2007; Miles & Huberman, 1994; Yin, 1994).

Given the explorative nature of the research questions and the uniqueness of the COVID-19-induced crisis, employing a qualitative research design is particularly suitable and commonly applied (see e.g., Kraus et al., 2020a; Li et al., 2017; Metzler & Muntermann, 2020). It is the preferred strategy "when the investigator has little control over events, and when the focus is on a contemporary phenomenon within some real-life context" (Yin, 1994, p. 1), as it is the case in our study context: Even though the theoretical background showed that extensive research on ICT-enabled BMI and ICT adoption exists (see Section 2), the circumstances of the COVID-19 pandemic and the related guidelines on physical distancing are unprecedented. Accordingly, a qualitative research methodology is used that is both, deductive and inductive, and aims to generate, elaborate and, test theories in order to extend existing theories (Kraus et al., 2020a; Lee et al., 1999). More specifically, the qualitative approach is applied to test and extend existing research on ICT-enabled BMI as a strategic crisis response, and on ICT adoption as a measure, to overcome physical distancing. To achieve this, we build upon previous research by adopting the TOE framework and technology-affordances-and-constraints theory as initial guides for the data collection and iterative data analysis (Eisenhardt, 1989; Walsham, 1995).

### Sample and Data Collection

We draw on purposive sampling, a non-random sampling technique that allows for deliberate participant choice based on the researchers’ assessment of potential participants’ knowledge or experience, as well as the participants’ willingness to contribute (Etikan et al., 2016; Kraus et al., 2020a; Li et al., 2017). Accordingly, we approached SMEs in the business event industry that provide primarily business-to-business (B2B) and in some cases, also business-to-consumer (B2C) services. We detected no crucial differences between SMEs with B2B focus compared to SMEs that serve B2B as well as B2C customers, so that we do not distinguish between them in the analyses.

We constrained our sample to Germany-based SMEs, as the business event industry is dominated by local players and the severity and the timeline of the pandemic differed between countries. This also applies to measures that were taken by governments to manage the crisis. Germany was first confronted with COVID-19 cases in March 2020 and went straight into lockdown, leading to declining rates of infection and relaxation of measures during the summer months (Worldometer, 2021). The first wave of infections was followed by a second, more severe one, starting in October 2020 which resulted in another lockdown of businesses and restrictions on public and private gatherings for several months (FAZIT Communication, 2021). In addition, Germany has particular relevance in the global business event industry, as it ranks second worldwide in terms of hosting conferences and fairs (ICCA, 2020). Every year, around 180,000 exhibitors and 10 million visitors spend a total of 14.5 billion euros on trade show activities in Germany, making the event industry the sixth largest economic sector in Germany, employing more than 1.5 million people (Feuerbach et al., 2020).

All interviews were conducted in November and December 2020 and therefore during the second lockdown in Germany. We selected our interview partners by screening the web for German-based SMEs in the business event industry that were already considering different ICTs (e.g., video-conferencing tools or virtual and augmented reality approaches) in their portfolio. Most of the interviewed SMEs focus on offering B2B events and were used for replication and extension reasons as well as to eliminate alternative explanations (Eisenhardt & Graebner, 2007; Yin, 1994). Moreover, half of the approached SMEs offer B2C solutions in addition to their B2B services which enabled analysis of diverse perspectives and for bias limitation (Eisenhardt & Graebner, 2007). Details on each interviewee’s SME as well as on the interviewee’s position can be found in Table 1.

**Table 1:** Overview of interviewees and their SMEs

ID	Event industry focus	Customer focus		Permanent employees (+ freelance staff)	Revenues in 2019 (rounded, in kEUR)	Interviewee’s position
		B2B	B2C			
E1	• Location owner • Event agency	x	x	14	350	Managing director, co-owner
E2	• Event agency • eCommerce	x	x	40	8,000	Team manager
E3	• Event agency • Congress/conference organizer	x		5	500	Project manager, co-owner
E4	• Fair/exhibition organizer	x	x	5	250	Managing director, artistic manager, co-owner
E5	• Live-communication agency • Fair/exhibition organizer	x		5	750	Managing director, co-owner
E6	• Event organizer	x	x	16	100	Project manager
E7	• Live-communication agency • Technology and solution provider	x		2 (+ 14)	150	Owner, co-founder
E8	• Live-communication agency • Congress/conference organizer	x		12 (+ 12)	3,000	Managing director
E9	• Live-communication agency • Congress/conference organizer	x		40 (+ 15)	2,000	Managing partner
E10	• Fair/exhibition organizer	x	x	4	250	Project manager

All interviews were conducted as semi-structured interviews which allowed for spontaneous reactions of the interviewer based on the interviewees’ statements (Myers & Newman, 2007; Neergaard & Ulhøi, 2007). We guaranteed interviewees’ full anonymity and offered them a report of the results. Due to physical distancing regulations, we conducted nine interviews via video-call, and only one face-to-face. Our interview guide followed the TOE framework’s three dimensions in an adjusted order. It contained a set of scripted questions that provided an overall guidance for the conversation but allowed for openness and flexibility (Myers & Newman, 2007). More specifically, the guide comprised questions including specific questions on the SME’s organizational structure and working model, on perceptions regarding the environmental dimension and COVID-19-related changes, and on the SME’s adoption of ICTs before and after the outbreak of the COVID-19 pandemic. Particularly with respect to the technological dimension, we attempted to obtain an accurate, undistorted view of the current situation by adding questions on specific and tangible ICT implementations, as well as current and potential future use cases. Guiding questions from our interview guide which were slightly modified or deepened over the course the interviews can be found in Table 4 in the appendix.

### Data Analysis

**Fig. 2:**
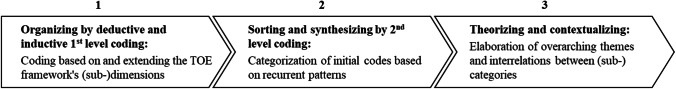
Analysis technique applied to qualitative data from semi-structured interviews

We audio-recorded and fully transcribed all interviews (average length of 40 min) by following the transcription rules of a simple transcript (e.g., omission of non-verbal communication elements, approximation of dialect to standard language) (Dresing et al., 2015). Subsequently, two researchers coded the transcripts independently to avoid misinterpretation or coding bias. Interviews were analyzed with systematic coding procedures, using MAXQDA software, a commonly used tool to assist the coding and analysis process (Kuckartz, 2014; Mayring, 2014). We built on established recommendations for qualitative data analysis and applied both, deductive and inductive coding in a hybrid coding method (Saldana, 2013). To do so, a provisional list of initial codes was determined beforehand based on the TOE framework’s dimensions and sub-dimensions (deductive) and extended in a data-driven first-level coding procedure (inductive). We proceeded with second-level coding by clustering codes into categories and sub-categories according to recurrent patterns (e.g., similarity), which were then linked with each other to elaborate themes and to investigate how they were interrelated, leading toward further refinement of our findings (Miles & Huberman, 1994; Saldana, 2013). Figure 2 shows the adopted analysis procedure.

Data analysis started after the first interview and continued after each interview until saturation was reached (i.e., no new insights are generated), which occurred after the ninth interview (e.g., Boddy, 2016; Eisenhardt, 1989; Morse et al., 2002). We conducted one additional interview to confirm that the termination criterion was achieved.

## Results

Our study reveals three key findings, structured along the three dimensions that affect ICT adoption in organizations (DePietro et al., 1990). Starting with the most affected dimension (i.e., environment), followed by the other dimensions (i.e., organization and technology), the insights within each dimension are presented by specifying and expanding their respective sub-dimensions (see Table 2 for an overview) and can be summarized as follows:

1. Environment: Disruptions in the environment create impetus for ICT-enabled virtual events
2. Organization: Building on their organizational strengths, SMEs can adopt ICTs to innovate their business model in response to the crisis
3. Technology: Readily available ICTs are clearly preferred over more sophisticated solutions for short and medium term BMI

Finding 1 and 2 provide insights that are required to answer our first research question. Both findings suggest that ICT adoption is an appropriate and promising crisis response strategy for SMEs in the business event industry to address the COVID-19-induced crisis. On the one hand, the crisis caused disruptions in the SMEs’ environment that fostered the need for and acceptance of ICT-enabled events. On the other hand, the organizational characteristics of SMEs facilitate the rapid innovation of their business model and thus, the adoption of ICTs in their value creation process. Finding 3 relates to our second research question by contrasting readily available and more sophisticated ICTs based on their affordances and by indicating that readily available ICTs are most promising for SMEs in the business event industry to adapt to the COVID-19-related physical distancing guidelines in the short and medium term.

**Table 2:** Summary of the study’s main findings along the dimensions environment, organization, and technology

Dimension	Existing thinking, including specifications for SMEs	Extension for SMEs in the business event industry during COVID-19
Environment	The environmental level is characterized by: • Governmental regulations • Industry characteristics and market structure (incl. consumer readiness) • Technology support infrastructure • Market uncertainty	We confirm that the environmental influences ICT adoption by demonstrating its impact in the context of COVID-19: • Governmental regulations on gatherings (e.g., physical distancing, wearing face masks in public) necessitate BMI and thus, exert a positive effect on ICT adoption • Customers’ increased health concerns and the lack of alternative events have a positive effect on customers’ openness to ICT-enabled virtual events—however, customer readiness (i.e., ability to use certain ICTs) is critical for successful development of ICT-enabled events • Access to (externally) available, affordable, easy to use technology support infrastructure exerts a positive effect on rapid ICT adoption and acceptance by the industry and its customers • Market and environmental uncertainty are extremely high due to the lack of certainty around the duration of the pandemic, putting significant pressure on SMEs in the business event industry
Organization	SMEs are characterized by: • Small size, focused scope • Limited financial resources, limited access to skills and capabilities (multiple-role identities) • Lean managerial structure, flat hierarchies, low formalization level • Efficient internal processes, fast decision-making, short communication channels, little legacy IT • Influential owner-manager role • Culture with high levels of trust	We build on the existing organizational characteristics of SMEs and their effect on ICT adoption by investigating the impact on the organizational dimension from environmental disruptions and thus, how both dimensions interact: • Governmental regulations and changing market needs in the SMEs environment cause a demand for adjusted business models and consideration of ICT-enabled events • High levels of financial insecurity can result in employee layoffs or hinders new hiring, and as such, negatively affects the SMEs’ size and access to skills • Lean managerial structures and low formalization levels positively affect fast decision-making which is required during environmental disruptions to stay competitive • An innovative culture, openness to changing market demands and flexibility in terms of product offerings are key enablers to adjust to disrupted market environments • The influential owner-manager role is a key advantage—under the condition that owner-managers are open to innovations and familiar with available ICTs
Technology	Technologies are characterized by: • Availability (internally, externally) • Characteristics (complexity, compatibility)	We confirm the relevance of technological characteristics on ICT adoption and unveil how they are affected by the environmental disruptions: • External availability is positively affected with more ICTs being offered by external providers • Complexity and compatibility are positively affected with more customers having the required infrastructure and skills to be supplied with ICT-enabled events • Moreover, ICT adoption is not purely dependent on the ICT’s availability and characteristics, but also on the affordances and constraints it offers to SMEs and their customers (see Table 3 for details)

### Finding 1—Environment: Disruptions in the Environment Create Impetus for ICT-Enabled Virtual Events

The environment that surrounds SMEs in the business event industry significantly influences ICT adoption, and has been disrupted by the COVID-19 pandemic. In all of our interviews, the substantial impact of COVID-19-induced governmental regulations on players in the business event industry was emphasized. Specifically, mandatory physical distancing, banning of gatherings, and regulations on national and international mobility caused a collapse in demand resulting in event postponements and cancellations. As a result, SMEs in the business event industry had to reconsider the way they hosted events, for instance by adopting ICTs, leading to a shift towards virtual events in the market.

With regard to industry characteristics, we observe a predominance of small players, high seasonality of events, and the lack of ability to make up the revenue for cancelled or postponed engagements. Nevertheless, the interviews reveal that despite the challenges the business event industry is facing, its key players are confident that events will always play a central role in social and business contexts.

Moreover, we observe substantial changes in the business event industry’s market structure and customers. Players in the business event industry confirm that their suppliers and stakeholders (e.g., service providers, location owners) experience similar financial and existential challenges as themselves. In addition, the previously mentioned physical distancing guidelines inhibit stakeholder and customer interaction:

*"It’s also a challenge that now, when you give presentations to find new customers, it’s much more difficult to communicate on a personal level. We are still in the ‘people business’, where having the right chemistry plays a considerable role" (E5).*

Still, after a first wave of cancellations during the early stage of the pandemic, customers showed increasing interest in new, ICT-enabled events as an alternative for in person events. In this context, not only existing customers switched to new formats, but new customers could be acquired, and offering ICT-enabled events became essential to staying competitive in the market:

*"We have found new customers precisely because we now have this technology [virtual meeting and conference rooms] and are actively marketing it" (E5).*

For most customer segments, the trend towards more openness for ICT-enabled events is observed. Previous research showed that external shocks like the COVID-19 pandemic forced even resistant and late-adopting employees and managers to familiarize themselves with ICTs which provides such ICTs the opportunity to demonstrate their advantages once they are adopted (Kraus et al., 2020a). Insights from our interviews confirm this indication even though interview partners agree that the effect has not reached its full potential. The main reason is customer readiness which is described as heterogeneous, yet, critical for successful ICT adoption. Specifically, in settings where employees with different skill levels are required to join a virtual event (e.g., corporate event, conference), resistance is either feared by management or observed during events.

The interview partners agree that there was a comparably low level of digitalization of the technology support infrastructure within the business event industry prior to the outbreak of the pandemic (e.g., partially instable internet services or teams not equipped to connect virtually). The outbreak of the pandemic changed these circumstances for the better with new, affordable service offerings for ICTs becoming available.

The lack of plannability is a frequently referenced phenomenon of the COVID-19 pandemic, increasing market uncertainty. Crises are known for their sudden and large impact as they are triggered by surprising and disruptive causes (Pearson & Clair, 1998; Williams et al., 2017). However, the previously described effects of the crisis on the business event industry’s environment are not only of high impact but also have a low level of plannability with respect to their duration. Our interviews reveal a particularly strong perception of this pandemic as one consisting of a sustained lack of plannability. Even one year after the first COVID-19 cases were detected, there was still uncertainty about the pandemic’s course and sustainability of measures that were taken (e.g., unknown duration of lockdowns, uncertain mutation coverage by vaccination). Therefore, the environmental factors are even less controllable and make it practically impossible for individual SMEs to plan ahead. As one interviewee posits:

*"The uncertainty made the whole situation difficult. It was impossible to plan how long this Corona situation would last. Are we talking about three months? Are we talking about six months?" (E2).*

Due to these disruptive environmental changes, we find overarching optimism for long-term acceptance of hybrid events. Across all our interviews, there is an agreement that even though most events are forced into virtual formats right now, the future will not go back to purely physical events, but rather be dominated by ICT-enabled hybrid events. Such events combine the positive aspects of both, virtual events (e.g., greater range of audience, less travel efforts, higher flexibility) and physical events (e.g., deep personal exchange, informal interactions, emotional involvement):

*"We call it a smart interlink between the analog and virtual worlds […] In the future, you will always have a virtual extension, supplement, and expansion in the sense of a hybrid approach" (E9).*

However, the industry is still in its infancy with respect to adopting ICTs to ensure a seamless transition between both worlds. Interestingly, a switch to purely digital events once the battle against the pandemic is won and restrictions are lifted for good is considered unlikely as ICTs are not able to fully replace real-life interaction between people:

*"I don’t believe that the future lies in purely virtual events like watching virtual soccer or concerts. For me, that’s always just an add-on. […] An event is, after all, a social act" (E4).*

### Finding 2—Organization: Building on Their Organizational Strengths, SMEs Can Adopt ICTs to Innovate Their Business Model in Response to the Crisis

Based on the first finding and therefore, on the trend that the business event industry’s environment will continue to create impetus for virtual events in the short run and hybrid events in the long run, innovating an enterprise’s business model through ICT adoption is a promising strategic response for SMEs in the business event industry. Insights from our interviews confirm the existence of organizational challenges that SMEs typically face due to their small size: Human as well as financial resources are limited and as such, employees often have multiple-role identities and SMEs usually have a smaller asset base than larger enterprises. Our interviews reveal that in addition to these known challenges, and also as a consequence of limited human and financial resources, SMEs lack a dedicated innovation department and have less capital and resources to opt for a trial and error mindset. As one interview partner comments:

*"SMEs cannot always be as open to many things as large companies, which can easily try things out or have departments that take care of innovations" (E6).*

This challenge became even more pressing since the outbreak of the pandemic and the increase in pressure to rapidly reinvent one’s business model. Moreover, SMEs have more difficulties in finding employees with the relevant skills and also in promoting their new solutions as their marketing is not as sophisticated as it is for most larger enterprises.

However, there are some organizational advantages of SMEs that can offset their weaknesses when SMEs in the business event industry use them with foresight. These strengths comprise flat hierarchies, short communication channels, fast decision-making, and high levels of trust. Each of them is of great importance when it comes to ICT adoption in uncertain times. One interview partner highlights this aspect as follows:

*"We are good at reacting quickly and flexibly. We don’t have fixed structures and slow trains of thought that we have to stick to, we rather adapt right away" (E2).*

The same interview partner describes how employees were quickly discharged from their previous area of responsibility (e.g., research on physical event locations) and assigned to tasks more urgently required to innovate the SME’s business model and to adopt ICTs (e.g., evaluation of platform and software solutions for virtual events). Such agility requires an open-minded and trust-based culture, leveraging the benefits of multiple-role identities (i.e., broad skill-set with in a small team), but also certain managerial and leadership characteristics. Most interview partners report the existence of the earlier described influential owner-manager role who is often the prime decision-maker on operational and strategic level, and responsible for the SME’s survival. In multiple interviews we were able to verify the importance of managerial skills, IT and IS knowledge, strategic outlook, and a positive attitude for long-term success of SMEs, which in our case, is characterized by successful ICT-enabled BMI. Even though interviews revealed that many owners and managers found themselves confronted with existential fears during the early stage of the pandemic, they were able to overcome their state of shock and went immediately into constructive problem-solving. Such entrepreneurial spirit is a key requirement on the managerial level for successful ICT adoption and BMI. In this regard, it is not surprising that many interview partners describe themselves or their managers as individuals with an affinity for innovation and technologies, showing an intrinsic motivation to reinvent their businesses whenever it is required. One interview partner describes it as follows:

*"That is what drives us: We always have our finger on the pulse, we know exactly where the journey is going and are always at least two steps ahead of our customers" (E9).*

### Finding 3—Technology: Readily Available ICTs are Clearly Preferred Over More Sophisticated Solutions for Short and Medium Term BMI

Based on our interviews, we distinguish between *readily available* and *more sophisticated ICTs* when evaluating their adoption potential by SMEs in the business event industry. To this end, we build upon the availability and technological characteristics of both types of ICTs and outline their respective *affordances and constraints* for SMEs in the business event industry and for the business event industry’s customers.

Across all our interviews we identify predominantly use cases that are based on commonly used video-conferencing software and virtual meeting platforms (e.g., Zoom, Microsoft Teams) and leveraged for events that rely on live-streaming (e.g., conference talks), presentations (e.g., marketing events), or moderation of games and quiz-shows (e.g., team events). We summarize these solutions as *readily available ICTs* as they have already been market-ready and broadly available prior to the outbreak of the pandemic. Such use cases were implemented quickly in the early stages of the pandemic, after the first lockdown was announced, and have since been enhanced. One interview partner describes it as follows:

*"We sat down immediately [in April 2020] and evaluated what we could do remotely and online, and very, very quickly we released formats to the market that work completely from the home office" (E2).*

Most interview partners also describe potential future or already piloted use cases that comprise augmented or virtual reality. We summarize these solutions as *more sophisticated ICTs* because even though these ICTs are not completely new to the market, they are not as commonly used as the previously described readily available ICTs. However, our interviews reveal pilot use cases in which event participants use virtual reality glasses to attend team events (e.g., virtually travelling the world) and fairs (e.g., visiting exhibitors and their virtual stands). In addition, virtual reality meeting rooms can be provided to teams that work remotely or for interaction and networking at conferences. Moreover, other use cases enable participants to access the event via their web-browser or mobile phones to compensate for lacking hardware (e.g., virtual reality glasses). However, most interview partners agree that these use cases have not been implemented on large scale thus far:

*"We realized relatively quickly that the topic [virtual reality] is not yet suitable for the mass market, but rather for selected business-to-business areas, such as instructions and trainings" (E9).*

**Fig. 3:**
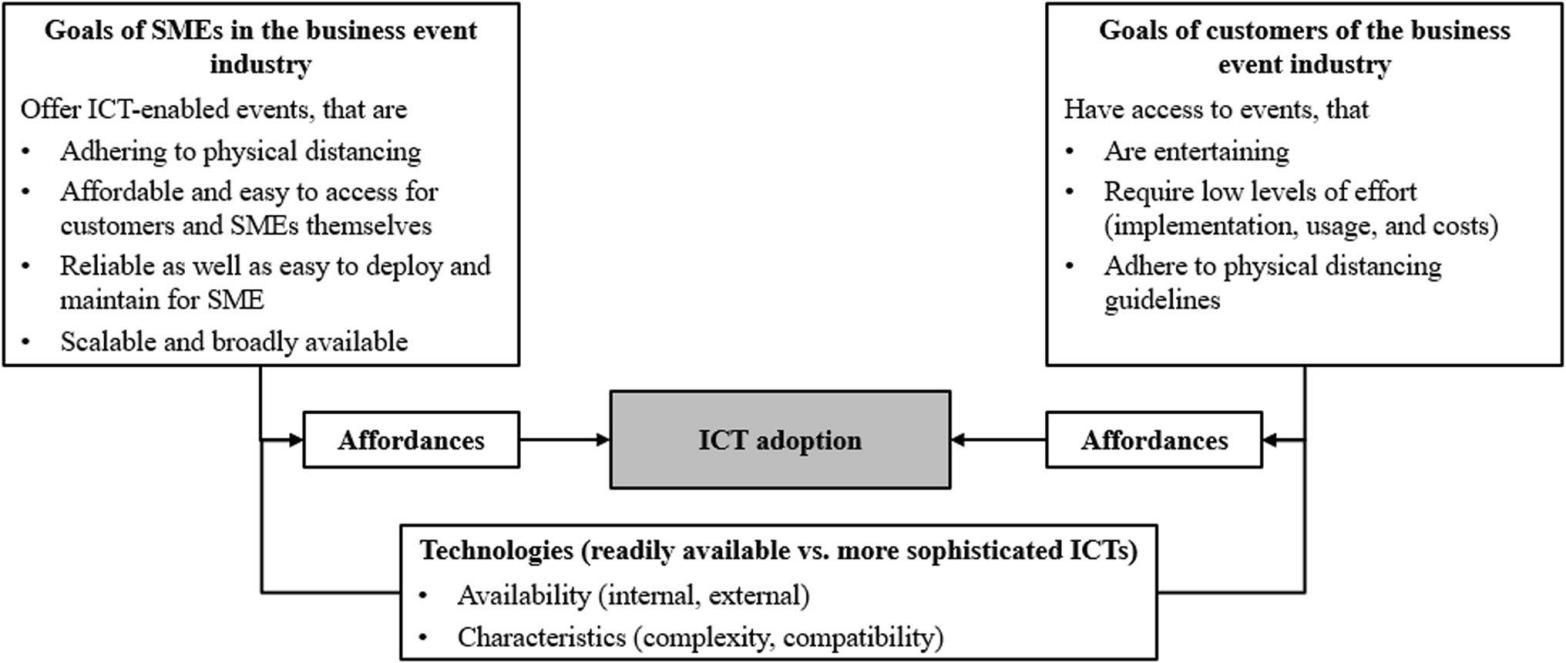
Goals of SMEs in the event industry and their customers with regard to ICT adoption during the COVID-19-induced crisis

As such, in line with previous research, an ICT’s internal and external availability as well as its characteristics (i.e., complexity and compatibility) have a significant impact on ICT adoption. However, whereas most scholars focus on organization-internal criteria for ICT adoption, we investigate ICT adoption in the context of virtual events offered by SMEs in the business event industry *towards their customers* which requires consideration of both stakeholder groups. This already points to the different ICTs’ affordances that a certain technology (i.e., readily available vs. more sophisticated ICTs) represents for an organization (i.e., SMEs in the business event industry) or individuals (i.e., event participants as the business event industry’s customers) with specific goals and characteristics. In the context of this study, the primary goal of SMEs in the business event industry in times of the COVID-19 pandemic is being able to offer events that allow participants to attend an event while simultaneously adhering to physical distancing guidelines. In addition, they want to bring affordable, reliable, as well as easy deployable and maintainable ICT-enabled events to the market to enable themselves to innovate their business models in the short and medium term. In addition, SMEs are looking for ICTs that allow for easy scalability and broad availability. Findings from our interviews reveal that players in the business event industry rely on ICTs that can support events with a wide range of participants, referring to a possibly high amount, as well as geographical spread, of participants. Event participants, on the other hand, are rather heterogeneous in terms of technology readiness and have different skill levels when it comes to ICT usage. However, their goals are to be well entertained at a low level of effort with regard to implementation, usage, and costs while being protected from the spread of the COVID-19 virus. These partially overlapping, partially differing goals, which are illustrated in Fig. 3, lead to different affordances offered by readily available versus more sophisticated ICTs for both stakeholder groups (see Table 3 for an overview) and consequently, impact ICT adoption.

**Table 3:** Affordances of readily available and more sophisticated ICTs

ICT	Affordances (AF) for…	
	… SMEs in the business event industry	… customers of the business event industry (i.e., event participants)
Both categories	1. Access to a global market with customers all around the world	2. Decentralized interaction with participants around the world
	3. Access to environmental-friendly events due to less travel and catering 4. Development of personalized events at a suitable level of effort	
Readily available ICTs (Video-conferencing software and web-based communication platforms)	5. Fast development and scaling of new events that comply with physical distancing guidelines 6. Access to reliable external support offerings and in-house expertise 7. Flexibility and readiness for potential future pandemic-related measures (e.g., another lockdown on short notice) or other crises	8. Easy access to new events that comply with physical distancing guidelines at low efforts and costs for installation and use 9. Entertainment with a high compatibility with the required ICT-related skill set 10. Reusable content that can be accessed flexibly (recordable sessions)
More sophisticated ICTs (Virtual and augmented reality)	11. High market entrance barriers due to high costs, efforts, and required expertise 12. Development of a greater variety of events to differentiate from competitors	13. Access to immersive events that allow for networking on an emotional level while complying with physical distancing guidelines 14. New and a rather unusual experience

Both, readily available and more sophisticated ICTs, provide SMEs in the business event industry with the affordance of having access to a global market with potential customers all around the world (affordance (AF) 1, Table 3). In addition, they provide event participants with the affordance of decentralized connections and interactions with other participants around the world (AF 2), a notable affordance, also beyond the COVID-19 pandemic:

*"Decentralization will still be in demand [post-crisis] and will mainly come from companies that have several locations. Whenever we want to involve multiple locations or countries, online events will continue to be in demand" (E2).*

This is also related to the finding that SMEs and participants are both provided with the affordance of accessing sustainable events that are environment-friendly because no travel or catering is required (AF 3). Moreover, both benefit from the affordance of developing personalized events at a suitable level of effort, for instance by incorporating the customers’ corporate identity into the event layout (AF 4).

For events that are based on readily available ICTs, we find that such solutions provide SMEs in the business event industry with the affordance of fast development and scaling of new events that comply with physical distancing guidelines and that come at a reasonable effort and price (e.g., for software, hardware) as they rely on existing or easy to install infrastructure (AF 5). In addition, as readily available ICTs are commonly used, SMEs in the business event industry have access to reliable external support offerings and in-house expertise and consequently, do not need to upskill their employees (AF 6). Moreover, such ICTs afford flexibility and help SMEs in the business event industry to be prepared for future pandemic-related measures (e.g., another lockdown on short notice) or other crises and market disruptions (AF 7). One interview partner highlights this aspect as follows:

*"Maybe there will be another virus, the next pandemic. In the future, you always must have a plan B, which means you have to know what happens if the event is canceled only two weeks in advance" (E9).*

For event participants, events that leverage readily available ICTs afford easy access to new events that comply with physical distancing guidelines and require low efforts and costs for installation and use (AF 8). Moreover, event participants can obtain entertainment in a way that is highly compatible with the average participant’s ICT-related skill set (AF 9). As most people already use such readily available ICTs to communicate in their private or business lives, event participants are often familiar with these ICTs:

*"The good thing about Microsoft Teams is that very many companies in Corona times already use it and therefore, many people already know the program and get along very well with it, which is why we very quickly got into this program" (E2).*

Finally, events that rely on readily available ICTs can usually be recorded and, as such, provide event participants reusable content that can be accessed and reused flexibly according to the individual’s requirements (AF 10). As one interview partner comments:

*"Sustainability is meant also in the sense of content. Preparation is sustainable, because the content that exists as a result of a virtual event is sustainably available and not simply gone like a real event" (E9).*

Nevertheless, readily available ICTs also have constraints which arise from the absence of desired affordances and may hinder implementation. Such desired, yet not offered affordances for SMEs in the business event industry would enable existing SMEs to differentiate themselves and to create market barriers to entry for new players as well as to offer a diverse range of events. Neither is sufficiently provided by readily available ICTs as competitors and new entrants benefit from the identical advantages of these solutions (i.e., low effort and cost for software, hardware, and support). In addition, the downside of developing events that meet the average participant’s ICT-related skill level limits the variety of offered events. From the event participants’ perspective, readily available ICTs that offer mainly voice- and video-transmission in combination with basic participant interaction (e.g., screen sharing, annotate tools, emojis for emotional reaction during a video conference) have the constraint of not affording an emotional interaction that is comparable to real-life interaction.

More sophisticated ICTs, on the other hand, can provide these affordances when they are properly implemented. For SMEs in the business event industry, sophisticated ICTs provide the affordance of a high market entry barrier that makes it more difficult for new players to enter the market due to comparably high costs, efforts, and required expertise (AF 11). Furthermore, events that rely on more sophisticated ICTs offer the possibility for SMEs in the business event industry to develop a greater variety of events which allows them to differentiate themselves more strongly from competitors (AF 12). Event participants have different goals and therefore different affordances that are related to events that rely on more sophisticated ICTs. For them, these ICTs provide the affordance of accessing immersive events (AF 13). This means that event participants can access events that allow for networking and exchange on a highly emotional level that is more comparable to physical interactions. Thereby, the previously mentioned constraint of readily available ICTs is addressed where participants can only communicate on a transactional level. The more an event is based on exchange of emotions (e.g., networking events, festivals, and sport events), the more important immersion and emotional involvement becomes and the more pronounced are the affordances of sophisticated ICTs. Furthermore, event participants can experience new and rather unusual events which afford high levels of satisfaction for participants looking for diversified entertainment and events that appeal to many senses (AF 14). For instance, while front seats in a physical event are limited and do not even exist in a video-conference, many participants of a virtual reality event can be provided a first row experience.

Nevertheless, more sophisticated ICTs also come with constraints. Due to their sophistication and less experience with their implementation, more sophisticated ICTs cannot provide the affordance of easily accessible and reliable support and more often lack experience with incident management. As a result, concerns about security and privacy incidents can occur. Moreover, most SMEs in the business event industry do not have the required in-house expertise and therefore experience the constraint of being dependent on external providers. From the perspective of event participants, events that rely on sophisticated ICTs cannot provide the affordance of easy and inexpensive access as participants that lack the required infrastructure (e.g., virtual reality glasses) or expertise (e.g., knowledge how to use virtual reality glasses) cannot attend an event in the intended way which then inhibits scalability of such events from the perspective of SMEs.

To summarize, perceptions of an ICT’s affordances encourage SMEs from the business event industry and event participants to engage themselves in events that rely on such an ICT, while perceptions of an ICT’s constraints can result in its rejection. Given the common usage of readily available ICTs by employees of SMEs in the business event industry, but also by potential event participants, readily available ICTs’ affordances are currently outweighing those of more sophisticated ICTs while readily available ICTs’ constraints are less of a hindrance than those of more sophisticated ICTs. In line with previous research that indicates that perceived barriers of an ICT have a greater influence on ICT adoption than its perceived benefits (Chau & Tam, 1997), readily available ICTs have the higher adoption potential by SMEs in the business event industry and their customers. Nevertheless, as familiarity with ICTs and the goals of the involved stakeholders evolve over time, more sophisticated ICTs’ affordances are likely to gain importance and in this way, become superior in the future.

## Discussion

The objective of our study was to answer the questions of how SMEs in the business event industry can leverage ICTs to address the COVID-19-induced crisis and what ICTs are most promising to adapt to physical distancing guidelines in an industry that has traditionally been dominated by physical interaction. Drawing on previous literature on ICT-enabled BMI and ICT adoption in organizations, we address these two questions. We find evidence for the feasibility of ICT adoption as a strategic crisis response for SMEs in the business event industry. Those SMEs that responded to the crisis successfully managed to overcome typical challenges of SMEs by leveraging commonly available ICTs to innovate their business models and service offerings as well as by building on SMEs’ strengths with regard to fast decision-making, short communication channels, and influential owner-managers. Furthermore, by applying technology-affordances-and-constraints theory to readily available as well as more sophisticated ICTs, our research advances our understanding on why readily available ICTs’ affordances and constraints outweigh those of more sophisticated ICTs to address the COVID-19-related physical distancing guidelines in the short and medium term. Besides, we outline how this might change in the long run.

## Research Contributions

We contribute to the existing literature on organizational ICT adoption and ICT-enabled crisis response strategies applied by SMEs in three important ways: First and foremost, we provide a more nuanced understanding of how ICT adoption by SMEs is influenced by the affordances and constraints of certain ICTs that can be leveraged to mitigate the detrimental economic impact of mandatory physical distancing in environments that traditionally rely on physical interaction. Previous research has investigated the role of ICTs (e.g., self-registration services in hospitals or social media) in facilitating digitization and overcoming societal or organizational challenges (e.g., institutional change, support of social movement organizations) (e.g., Essén & Värlander, 2019; Selander & Jarvenpaa, 2016; Volkoff & Strong, 2013). However, ICTs have usually been introduced as solutions that complemented rather than replaced physical interactions between people (i.e., employees and event participants). Furthermore, particular actors in different environments experience different affordances and constraints with the same ICT (Majchrzak et al., 2016). Therefore, by uncovering and contrasting the affordances and constraints of the most promising ICTs for SMEs and their customers in the context of physical distancing, we contribute to an enhanced understanding of the impact of such ICTs’ affordances and constraints on ICT adoption by SMEs.

Second, we extend the TOE framework by investigating how crisis-induced environmental disruptions impact ICT adoption in SMEs. The TOE framework covers a variety of internal (i.e., organizational) and external (i.e., environmental) factors as well as characteristics of ICTs that are crucial for successful ICT implementation in organizations (DePietro et al., 1990). However, the TOE framework has largely been applied in stable and relatively slowly changing environments, for instance, by looking at competition intensity and regulations in e-business environments (Zhu et al., 2004). Thus, the environment conditions have not made the significant effects on ICT adoption fully visible, such as in the case of market competition in SMEs’ environments and its effect on ICT adoption (Thong, 1999) or market uncertainty’s effect for open system adoption (Chau & Tam, 1997). We extend such previous research by investigating how the different aspects of the environmental, organizational, and technological dimension are impacted by the circumstances when a severe environmental disruption occurs, as has been the case with the COVID-19 pandemic. Moreover, with regard to the framework’s technological dimension, we extend the common approach of investigating technology characteristics and functionalities, such as technology readiness, compatibility, and complexity (e.g., Chau & Tam, 1997; Thong, 1999; Zhu et al., 2004). To this end, we contribute to research on ICT adoption in conjunction with the affordances and constraints that are provided by certain ICTs to SMEs and their customers. As such, by shedding light on the TOE framework’s dimensions in times of a crisis, we provide an enhanced understanding of the dimensions’ characteristics and interrelationships and contribute to a more encompassing view of the TOE framework in the context of SMEs.

Third, we contribute to knowledge on the extent to which ICT-enabled BMI can be considered a strategic crisis response for SMEs in the business event industry. Whereas previous research on strategic crisis response studied different crisis intervention measures and how they can be applied by enterprises of different sizes and industries (Kraus et al., 2020a; Wenzel et al., 2020), we contribute to this research stream by shedding light on the under-investigated, yet crisis-prone group of SMEs (Chan et al., 2020; Li et al., 2017). More specifically, we uncover how SMEs leverage readily available ICTs to innovate their business models despite the organizational and managerial challenges (e.g., lacking financial and human resources, multiple-role identities, geographical insularity) that they are typically facing. Therefore, we uncover important insights for crisis response strategy literature on how SMEs respond to the crisis, considering their specific characteristics.

### Practical Implications

Our results are of considerable interest for practitioners (i.e., executives of SMEs in the business event industry) who strive to leverage ICTs to innovate their business models in a way that allows for virtual events which adhere to physical distancing but also ensure customer acceptance. Whereas ICTs are usually advertised by their functionalities and benefits, previous research indicated that perceived barriers of an ICT have a greater influence on ICT adoption than its perceived benefits (Chau & Tam, 1997) and that such perceptions differ for different actors (Majchrzak et al., 2016). Thus, executives of SMEs in the business event industry are well advised to evaluate available ICTs not only with respect to affordances (i.e., perceived benefits such as broader range of customers or reduced efforts due to less travel activities), but also to constraints (i.e., perceived barriers such as lack of emotional interaction between customers or lack of reliable support offerings). Moreover, they are counselled to take their own as well as their customers’ perspective. By doing so on a regular basis, SMEs in the business event industry may be able to select appropriate ICTs for innovative events, which is required to emerge strengthened from the current crisis and to be well prepared to tackle any future crises.

Lastly, by highlighting the interrelation between an enterprise’s or an individual’s goal and their perceived affordances and constraints of different ICTs, our findings offer valuable insights for key players in the business event industry with regard to their long term success in the market. The previously described goals of SMEs in the event industry and their customers may change in the long run: As soon as physical distancing is not mandatory any longer, customers are likely to ask for an increasing share of (physical) interaction during events. Similarly, SMEs in the business event industry will aim to satisfy their customers’ needs, but also to differentiate themselves from their competitors and new market entrants. Considering these dynamically changing goals, SMEs in the business event industry are urged to reevaluate the affordances and constraints of more sophisticated ICTs regularly to strengthen their market position and to offer their customers immersive experiences. As such, our findings are valuable to support SMEs in the business event industry within and beyond the COVID-19-induced crisis.

### Limitations and Directions for Future Research

As every research, also our study is not without limitations. First, the results are based on a sample of expert interviews that is limited in size and located exclusively in Germany. Even though Germany is one of the leading countries when it comes to hosting business events, SMEs from different countries have experienced different impacts from the COVID-19 pandemic or have been impacted by other governmental regulations which may result in them perceiving and weighing the affordances and constraints of ICTs differently. Therefore, to improve generalizability of our findings, we call for future research to investigate ICT adoption by SMEs in the business event industry in different geographical contexts.

Second, our findings are based on interviews that took place during the second wave of infections of the COVID-19 pandemic. While our findings provide insights on the affordances and constraints of different ICTs in the short and medium term, we can only hypothesize how perceptions will change in the long run and whether different ICTs are preferred once the pandemic is under control and governmental restrictions are lifted for good. Thus, we encourage research to investigate whether and how affordances and constraints of readily available and more sophisticated ICTs change over time and how this impacts ICT adoption by SMEs in the business event industry.

Third, this study demonstrated the affordances and constraints of ICTs in their function to overcome physical distancing and to allow for virtual events that connect event participants whilst also allowing for physical distance. Once physical distancing regulations are released and hybrid or fully physical events are allowed, event participants might still be worried about the infection risk of highly infectious diseases in crowded spaces which are typical for business events. Hence, we encourage scholars to combine insights from our study with findings from research on ICT-provided crowding information (Adam et al., 2020) which could be a great measure to indicate participants of business events preference towards highly versus less crowded areas and as such, to minimize infection risks also with regard to future pandemics.

## Appendix

**Table 4:** Guiding interview questions for semi-structured interviews that were modified or deepened according to the respective interview partner

Key area	Guiding questions
(1) Enterprise/interviewee profile	Please provide a short description of your enterprise, its business model and your role
(2) Organizational dimension (SME)	What are typical characteristics of SMEs? From your experience, what are advantages and disadvantages of SMEs compared to larger enterprises?
(3) Technological dimension (ICTs)	What technologies, particularly ICTs, are used by your enterprise, internally but also with regard to customer interaction? How are new ICTs introduced to your enterprise? What are typical challenges or barriers when implementing ICTs? What are drivers for ICT implementation and adoption in your enterprise? What are use cases for virtual or augmented reality solutions? What are benefits of virtual and augmented reality compared to other ICTs? What are barriers for their implementation?
(4) Environmental dimension (market, industry, and COVID-19)	What are drivers for innovation and ICT adoption in your industry? How did COVID-19 affect your enterprise and the way it is operating? How did COVID-19 affect the market in which your enterprise operates and its key players? Did the competitive situation, customer segments or partnerships change? How did COVID-19 affect the adoption of ICTs by your enterprise’s environment?
(5) Future perspective	How do you think will the industry and also your enterprise be operating in two to five years? What ICTs do you expect to stay? What ICTs do you expect to become less relevant?

## Footnotes

1 Often referred to as “social distancing “. We refrain from using the term social distancing because ICT-based social interactions (e.g., video-conferencing) are not restricted by regulations related to the COVID-19 pandemic.
